# Preparation and Electrical Properties of Polyacrylonitrile Based Porous Carbon by Different Activation Methods

**DOI:** 10.3390/molecules26123499

**Published:** 2021-06-08

**Authors:** Xiaoqiang Wang, Yifan Tan, Meijiao Sun, Binbin Yu, Junhe Yang, Yuhua Xue, Guangzhi Yang

**Affiliations:** School of Materials Science and Engineering, University of Shanghai for Science and Technology, Shanghai 200093, China; XQ320557@163.com (X.W.); tyf285101725@163.com (Y.T.); 18307416856@163.com (M.S.); 15121197769@163.com (B.Y.); jhyang@usst.edu.cn (J.Y.); xueyuhua@usst.edu.cn (Y.X.)

**Keywords:** porous carbon, activation method, specific capacitance, retentions

## Abstract

Polyacrylonitrile (PAN)-based porous carbon was prepared by different methods of activation with PAN polymer microsphere as precursor. The morphology, structure and electrical properties for supercapacitor of the porous carbon were investigated. It was found that the morphology of PAN nanospheres tended to be destroyed in the process of one-step activation (activation and carbonization were carried out simultaneously, and could only be retained when the amount of activating agent KOH was small). While the spherical morphology could be well reserved during the two-step activation method (carbonization and activation sequentially). The specific surface area and pore volume increased first and then decreased, with the increase in activation holding time for both one-step and two-step activation methods. The specific surface area reached the maximum value with 2430 m^2^ g^−1^ for the one-step activation method and 2830 m^2^ g^−1^ for the two-step activation method. Additionally, their mass-specific capacitances were 178.8 F g^−1^ and 160.2 F g^−1^, respectively, under the current density of 1 A g^−1^. After 2000 cycles, the specific capacitance retentions were 92.9% and 91.3%.

## 1. Introduction

As an energy storage device, the supercapacitor has a wide range of applications in many fields, such as traffic vehicles, electronic products, green energy, and military fields [1,2,3]. Additionally, there are many types of electrode materials for the supercapacitor, including carbon materials, metal oxides, hydroxide materials, conductive polymers and so on [4,5,6,7,8,9,10]. Among them, carbon materials are widely used because of their high conductivity, low cost, and good chemical stability [11,12]. A variety of materials have been used as precursors to generate carbon materials, including polyaniline, polyacrylonitrile (PAN), aminopyrine and so on [13,14,15,16]. PAN is an available and inexpensive material which is widely used for preparing carbon materials with high yield and certain nitrogen group [17,18]. PAN-based carbon materials are usually prepared through the process of polymerization, oxidation, carbonization or activation. Different activation methods can be used for the preparation of PAN-based carbons: the one-step activation method combines the carbonization and activation simultaneously, while in the two-step activation method, carbonization occurs first and activation follows.

Lee, Byoung-min et al. prepared active porous carbon beads (APCBs) with polyacrylonitrile as raw material and KOH as activator. The prepared active porous carbon spheres have the specific surface area of 1147.99 m^2^ g^−1^. The APCB-based electrodes showed a good specific capacitance of 112 F g^−1^ at 1 A g^−1^ in a 6 M KOH electrolyte and excellent capacitance retention of 100% at a current density of 5 A g^−1^ after 1000 cycles [19]. Yao, Long et al. synthesized PAN based graded porous carbon nanospheres for high-performance supercapacitors by surfactant-free emulsion polymerization and one-step KOH activation method. The specific surface area and pore volume of the prepared porous carbon nanospheres were 3130 m^2^ g^−1^ and 1.87 cm^3^ g^−1^, respectively [20]. Liu, Yujing et al. prepared highly cross-linked porous carbon microspheres through carbonization and then KOH activation, and obtained a product with a specific surface area of 3065.6 m^2^ g^−1^. The optimal electrode presents the highest specific capacitance of 290 F g^−1^ and superior cycling stability with 96% retention after 3000 cycles in an aqueous 2 M KOH electrolyte [21]. Shu, Yu et al. used PAN as raw material and KOH as activator, prepared the N-doped porous monolithic carbons with a specific surface area of 1600 m^2^ g^−1^. The specific capacitance of 270 F g^−1^ at 0.2 A g^−1^ and 195 F g^−1^ at 100 A g^−1^. Additionally, it has a 100% capacitance stability at 20 A g^−1^ after 5000 cycles [18]. Chen, Guangpin et al. fabricated hierarchical porous carbon materials from PAN through phase inversion and carbonization. Additionally, supercapacitor assessments in 6 M KOH solution revealed a high capacitance of 156 F g^−1^ and 34.6 mu F cm^−2^ at 0.2 A g^−1^ [22].

In this paper, PAN-based porous carbon materials were prepared by different activation methods on the basis of soap-free emulsion polymerization. The morphology, structure and electrical properties of the porous carbon for supercapacitor were investigated under different methods of activation.

## 2. Results and Discussion

### 2.1. Two-Step Method of Carbonization and Activation Gradually

#### 2.1.1. Morphology Analysis

**Figure 1:**
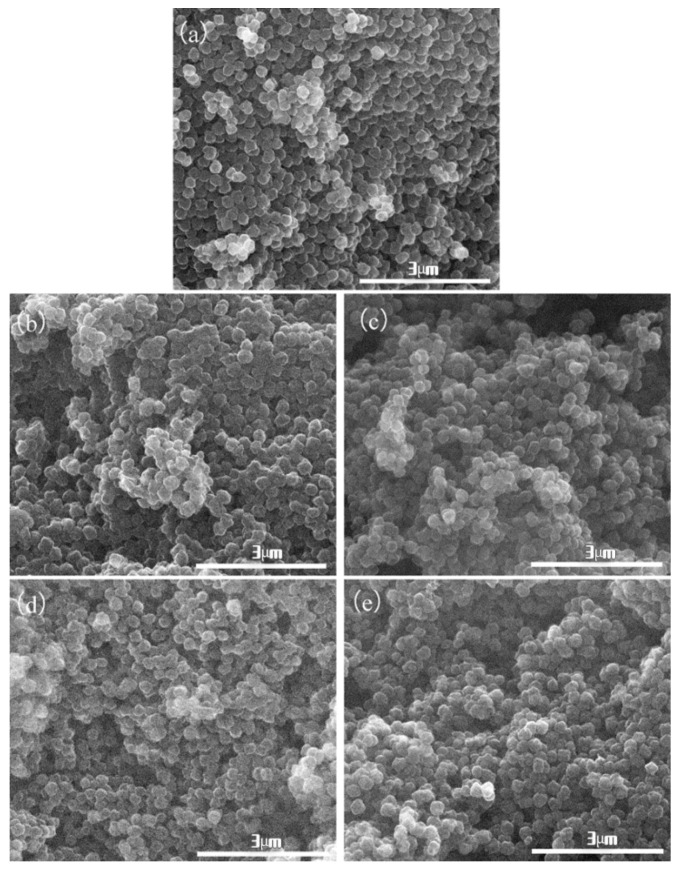
Material morphology of two-step activation method with different activation holding time: (**a**) CAPN; (**b**) 2APCNs-1; (**c**) 2APCNs-1.5; (**d**) 2APCNs-2; (**e**) 2APCNs-3.

Figure 1 shows the morphology of porous carbon materials with different activation holding time at the same KOH and CPAN mass ratio of 3:1. Figure 1a is the CPAN sample without activation. In Figure 1, it can be seen that the prepared carbon materials have a spherical morphology with the diameter of about 200 nm. Additionally, there is no obvious different appearance among the products of different activation holding time. That is, the spherical morphology of PAN polymer is retained in the process of carbonization and activation.

**Figure 2:**
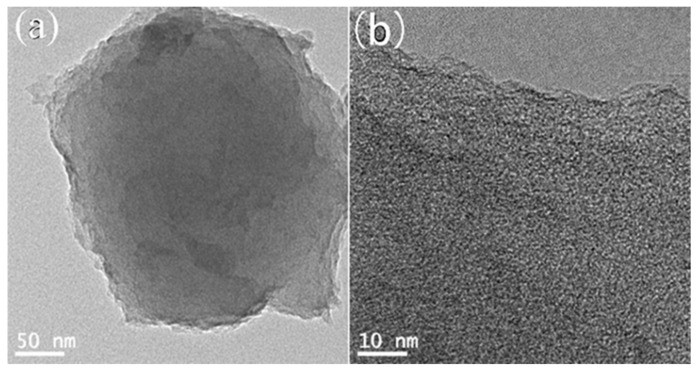
TEM image of 2APCNs-1: (**a**) Low magnification TEM image; (**b**) High magnification TEM image.

Figure 2 is the TEM characterization of the porous carbon of 2APCNs-1. A well-developed pore structure composed of worm-like micropores and small mesopores could be seen. It shows that the porous carbon material was successfully prepared by the two-step activation method. In Figure 2a, although the carbon spheres prepared by the two-step activation method showed spherical morphology, the edges of the sphere are not smooth and complete, showing irregularities. This indicates that KOH corroded the material, resulting in partial cracking and some damage of the sphere wall.

#### 2.1.2. Elemental Analysis

Table 1 is the elemental analysis results of the 2APCNs with different activation holding time. It can be seen that the carbon, nitrogen and oxygen contents of two-step activation method are approximately 82.7−89.18%, 4.05–5.56% and 6.22–11.74%, respectively. Additionally, the ratio of O/C is about 7.0–14.2%. When the activation holding time is 2 h, the minimum O/C of 7.0 and the highest carbon content of 89.18% are obtained.

#### 2.1.3. Pore Structure Analysis

**Figure 3:**
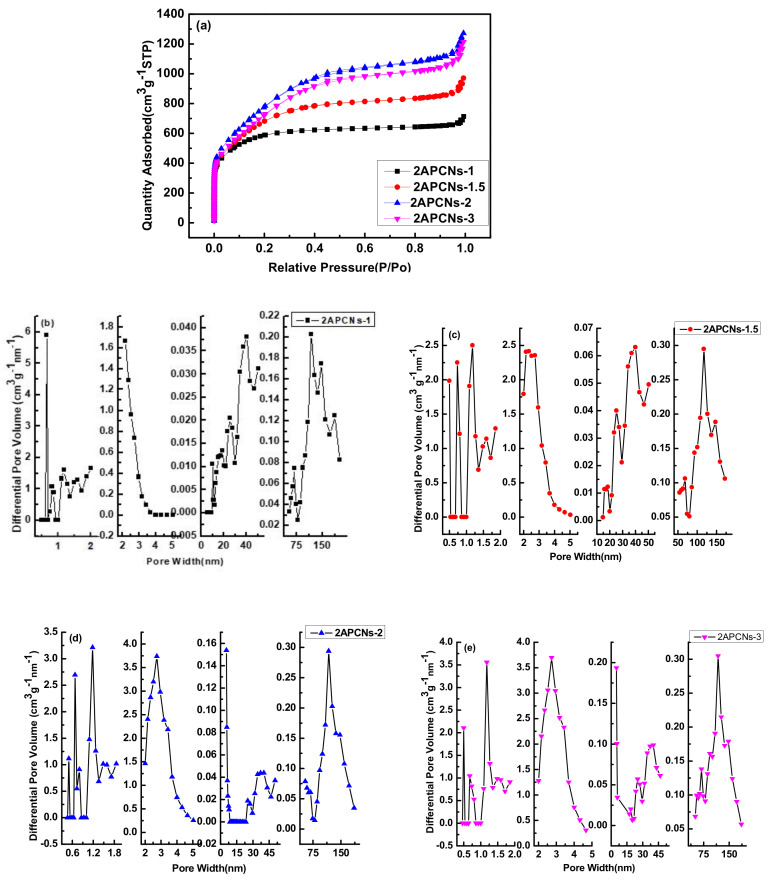
The adsorption isotherm curve and pore size distribution diagram of different activation holding time in two-step activation method: (**a**) adsorption isotherm curve of different holding time; (**b**) pore size distribution of 2APCNs-1; (**c**) pore size distribution of 2APCNs-1.5; (**d**) pore size distribution of 2APCNs-2; (**e**) pore size distribution of 2APCNs-3.

The Isothermal adsorption and diameter distribution curves of 2APCNs under different activation holding time by two-step activation method are shown in Figure 3. It can be seen that the activation holding time is an important factor affecting the pores of the material. The adsorption isotherm curve first increases and then decreases as the activation holding time increases. With the extension of the holding time to 2 h, the corrosion effect of KOH is enhanced, the micropores and mesopores formed are expanded, forming macropore or the pore volume disappears to form a bulk material.

Additionally, the adsorption isotherms of porous carbon materials under different activation holding times show different types. The saturation relative pressure of the sample 2APCNs-1 is about 0.2, and the curve shows a typical type I adsorption isotherm, indicating that the sample 2APCNs-1 is mainly composed of micropores. DFT method was used for the calculation of pore size distributions in this work. The maximum N_2_ adsorption capacity at a relative pressure around 0.99 is approximately 713.4 cm^3^ g^−1^, and the corresponding total pore volume is 1.14 cm^3^ g^−1^. When the activation holding time increases to 1.5, 2 and 3 h, the isotherm shows a typical IV type. Additionally, there is an obvious hysteresis loop in the relative pressure range of 0.4–1.0, indicating 2APCNs-1.5, 2APCNs-2 and 2APCNs-3 have a large number of mesopores with small diameter. In addition, in the relative pressure range of 0.95 to 1.0, the isotherm has a sharp rise, which indicates the existence of macropores. Figure 3b shows that the pore volume of 2APCNs-1 larger than 2 nm is very low. For 2APCNs-1.5, 2APCNs-2 and 2APCNs-3 with higher activation holding time, the sample has a significant increase in the pore volume of mesopores with a pore small diameter of 2–4 nm, as shown in Figure 3c–e.

BET method was used for the measurement of specific surface area, total pore volume, mesopore volume and diameter distribution in this work. Meanwhile, it can be seen from Table 2 of pore characteristics that the specific surface area, total pore volume, mesopore volume and diameter distribution of two-step activation method is about 1880–2830 m^2^ g^−1^, 1.10–2.19 cm^3^ g^−1^, 0.71–1.97 cm^3^ g^−1^ and 3.12–3.19 nm, respectively.

#### 2.1.4. Electrochemical Performance Analysis

**Figure 4:**
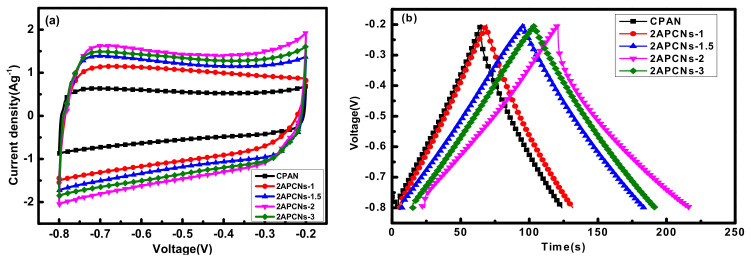
Electrochemical performance of each sample in KOH electrolyte: (**a**) CV curve with 10 mV s^−1^ scan rate; (**b**) Constant current charge and discharge with current density of 1 A g^−1^.

Figure 4 shows the cyclic voltammetry curves of the activated samples for 2APCNs and CPAN samples, respectively. As can be seen from the figure, the CV curve shows a rectangular-like shape, which is ideal for a double-layer capacitor. The specific capacitance of CPAN, 2APCNs-1, 2APCNs-1.5, 2APCNs-2 and 2APCNs-3 is 98.3, 104.5, 145.2, 160.2 and 145.7 F g^−1^, respectively. The results show that the electrochemical performance of the samples after activation has been improved, and the highest electrochemical performance is shown when the activation holding time is 2 h, indicating that specific surface area benefits the electrochemical performance.

**Figure 5:**
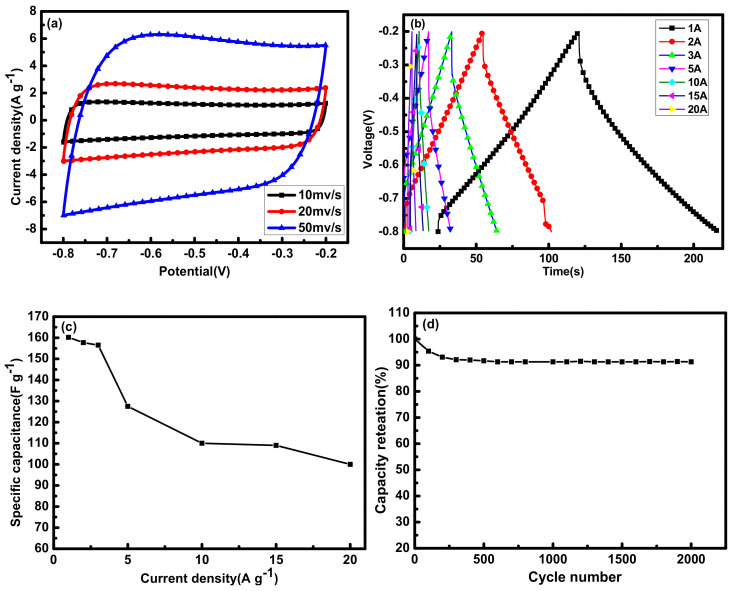
Electrochemical characterization of sample 2APCNs-2: (**a**) CV curve at different scan rates; (**b**) Constant current charge and discharge curve with current density from 1 A g^−1^ to 20 A g^−1^; (**c**) Line diagram of specific capacitance at different current densities; (**d**) Cyclic stability curve with current density of 1 A g^−1^.

The electrochemical performance of sample 2APCNs-2 was analyzed in detail. Figure 5a shows the CV curve with a scan rate ranging from 10 to 50 mV s^−1^. Even at a high scan rate of 50 mV s^−1^, the rectangular characteristic of the curve is still very good, indicating sample 2APCNs-2 has efficient charge transfer. In addition, Figure 5b is a constant current charge and discharge curve with a current density of 1 A g^−1^ to 20 A g^−1^, which also exhibits linear characteristics. Figure 5c is the specific capacitances under different current densities. As can be seen that with the current density increases, the specific capacitance decreases. Figure 5d is the cycle stability diagram of the 2APCNs-2 sample. At the current density of 1 A g^−1^, after 2000 cycles of the sample, the prepared electrode material maintains a specific capacitance of 91.3%; it shows that the sample has a good cycling stability.

### 2.2. One-Step Method of Carbonization and Activation Simultaneously

#### 2.2.1. Morphology Analysis

As mentioned above, the pore forming of carbon through activation in the two-step is carbonization and activation gradually. If carbonization and activation could be combined and carried out simultaneously in one step. The fabricating route would be simple and economic. Here, the method of one step is used for a comparison with the two-step method.

**Figure 6:**
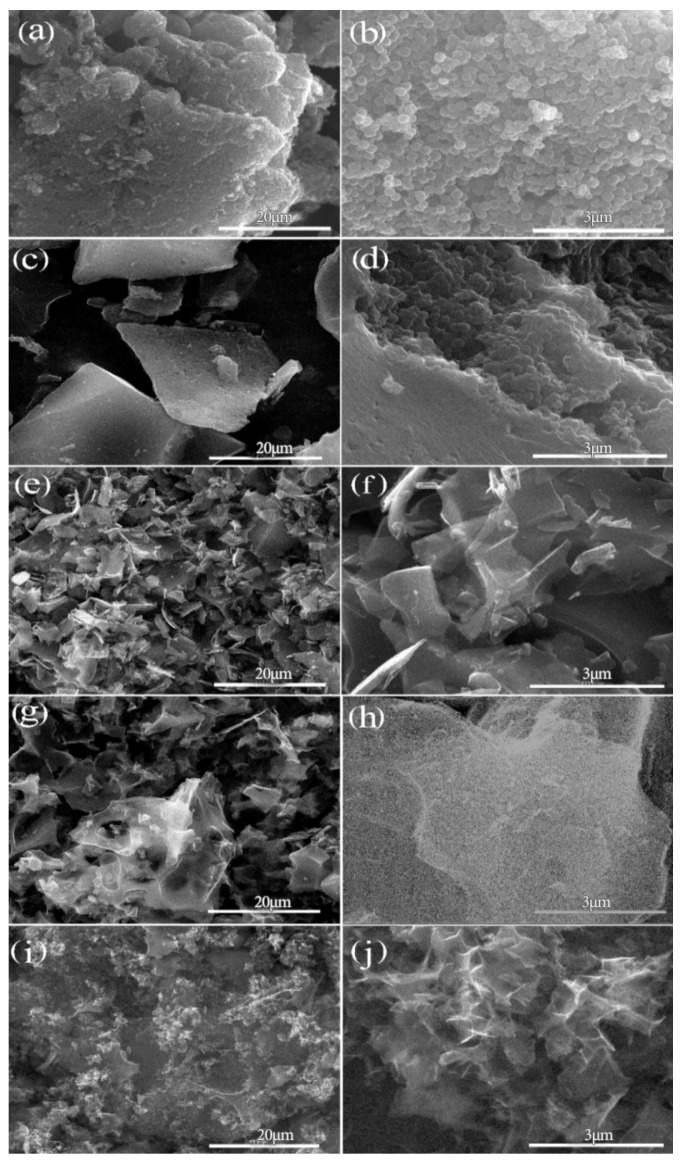
SEM morphology of different mass ratios of KOH and OPAN in one-step activation method: (**a**,**b**) APCNs0.05; (**c**,**d**) APCNs0.1; (**e**,**f**) APCNs0.5; (**g**,**h**) APCNs1; (**i**,**j**) APCNs3.

Figure 6 shows the SEM morphology of APCNs with different mass ratios of KOH and OPAN in one-step activation method. It can be seen that most of the products have non-spherical morphology except for the material of APCNs0.05. With the increase in alkali content, the corrosion damage degree of the material also increases. When the mass ratios of KOH and OPAN increases from 0.1:1 to 3:1, the morphology of the material also shows a certain rule: with the increase in mass ratio, the block size of the material first increases and then decreases, and the size reaches the maximum when the mass ratio reaches 1:1. When the mass ratio increases to 3:1, it can be seen from the SEM figure i, j that the material surface presents smaller irregular morphology. Because with the increase in alkali content, the corrosion on the surface of the material is strengthened. When the PAN spheres are destroyed at the beginning, the spherical radian is still small, so the material size is small. With the increase in alkali, the damaged radian of the PAN spheres becomes larger and the material size reaches the maximum. When the alkali content continues to increase, the alkali will seriously corrode the material and corrode and fracture the large-size material, resulting in the decrease in the material size. Moreover, because the corrosion of alkali has no direction, the size of the material is not uniform, forming an extremely irregular polygon morphology. Therefore, it can be concluded that mass ratios of KOH and OPAN have an effect on the morphology of the material. When the mass ratios of KOH and OPAN are lower than 0.05:1, the porous carbon material can maintain the spherical morphology.

**Figure 7:**
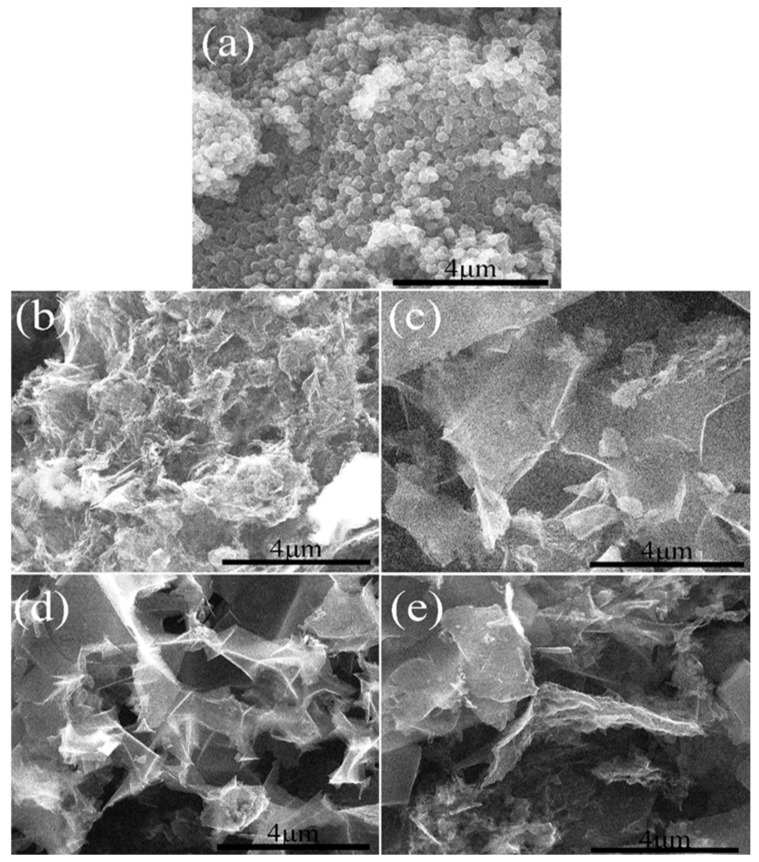
Morphology of one-step activation method materials: (**a**) CAPN; (**b**) APCNs-0.5; (**c**) APCNs-1; (**d**) APCNs-1.5; (**e**) APCNs-2.

Figure 7 shows the morphology of porous carbon materials with the same mass ratio of KOH and OPAN of 3:1 and different activation holding time. Figure 7a is the unactivated sample; it can be seen from the comparison between (a) and (b, c, d, e), different degrees of activation holding time will destroy the spherical morphology, with the increase in activation holding time, the size of the material decreases. Because with the extension of the holding time, the corrosion effect of alkali increases. When the holding time is 0.5 h, the corrosion effect of alkali is small, and the degree of material crushing is light. With the extension of the alkali action time, the material is further corroded in the formed block material, and the material is cut off, resulting in the size reduction. When the holding time reached 1.5 h, no significant difference was found in the material size, indicating that the activation holding time had the strongest effect at 1.5 h.

**Figure 8:**
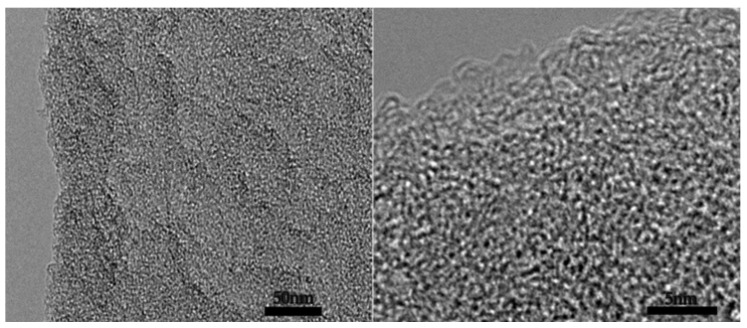
TEM image of APCNs-0.5.

Figure 8 shows a TEM image of APCNs-0.5. It can be seen from the figure that APCNs-0.5 have a developed pore structure with a large amount of worm-like micropores and mesopores. Among these pores, mesopores can provide fast electron diffusion channels to reduce the diffusion distance and micropores can enhance the electrical bilayer. These graded pores can improve the capacitive property of the material.

By comparing the one-step activation method with the two-step activation method, it is found that the two-step activation method could maintain the spherical morphology, because the two-step activation method first carried out the carbonization, and then the activation reaction was not intense, so the spherical morphology was not destroyed. However, the one-step activation method was used to carry out activation carbonization at the same time, and the surface reaction is more intense, which leads to the destruction of the spherical morphology.

#### 2.2.2. Elemental Analysis

Table 3 shows the elemental analysis of a series of APCNs products. It can be seen that the nitrogen content of the sample after activated carbonization still has a considerable amount, although it decreases greatly compared with that of un-activated sample CPAN. When PAN is oxidized, nitrogen atoms play a key role in the cyclization reaction of the material [23]. When the OPAN material is carbonized and activated, its molecular structure changes, carbon and oxygen content increases. Because the activator KOH brings a lot of oxygen elements into the material, and begins to produce acrylamide, HCN, NH_3_, CO_2_, propanol, acetamide, propanolamine and other substances, the element content also changes accordingly. At the same time, with the increase in the activation holding time, the O/C in APCNs also decreased first and then increased, and reached the minimum when the holding time was 1.5 h. This indicates that the highest carbon element is produced when the activation holding time is 1.5 h.

#### 2.2.3. Structure Analysis

**Figure 9:**
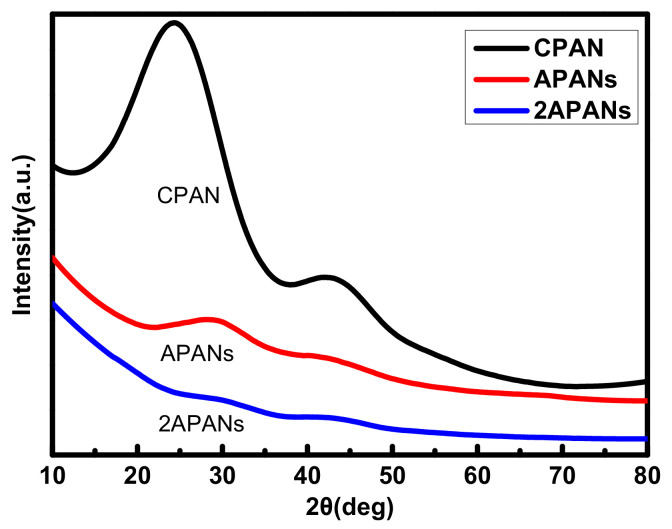
XRD patterns of different carbonized and activated materials.

Figure 9 shows the XRD patterns of different carbonized and activated materials. The peaks at 2θ ≈ 25° is weak for APCNs and even disappears for 2APCNs compared with that for CPAN, indicating that the degree of graphitization for the activated sample is reduced. Additionally, the same result is found about the peaks at 2θ ≈ 43°, indicating that the amorphous structure of the activated materials is more obviously existed. Because the strong etching of the material caused by activation, the activation reaction transfer gradually the carbon atoms to the benzene ring. Therefore, amorphous materials with a large specific surface area are gradually formed, and the graphitization degree is reduced.

**Figure 10:**
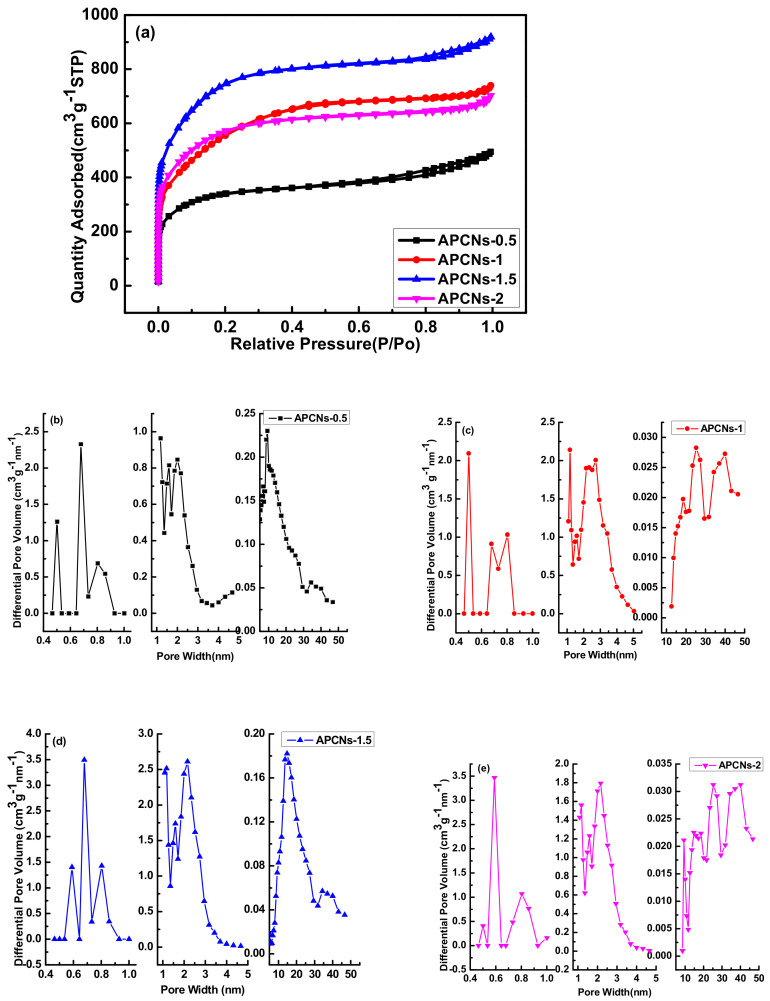
The adsorption isotherm curve and pore size distribution diagram for different activation holding time in the one-step activation method: (**a**) adsorption isotherm curve; pore size distribution for: (**b**) APCNs-0.5; (**c**) APCNs-1; (**d**) APCNs-1.5; (**e**) APCNs-2.

Figure 10 shows isothermal adsorption curve and pore size distribution of different activation holding time in one-step activation method. In the one-step activation method, the isothermal adsorption curve of porous carbon material at different activation holding time shows a typical IV type. Similar to the two-step activation method, different activation holding time in one-step activation method corresponds to different pore volumes, and each method has its own characteristics. Table 4 shows the pore characteristics of porous carbon materials prepared with different activation holding time in the one-step activation method. It can be seen that compared with the CPAN sample, the specific surface area of the activated samples increased greatly and large amounts of micropore and mesopore were formed. Activation holding time has a great effect on the pore structure of the materials. The specific surface area for APCNs-0.5 of 0.5 h holding time is 1080 m^2^ g^−1^ and increases to 2430 m^2^ g^−1^ by 2.25 times for APCNs-1.5 of 1.5 h. Additionally, the total pore volume increased by 1.87 times, reaching to 1.42 cm^3^ g^−1^. The mesopore volume also increased sharply, reaching to 1.14 cm^3^ g^−1^. The structural characteristics of the graded pores are good to ion diffusion and the surface contact between electrolyte and electrolytic material.

**Figure 11:**
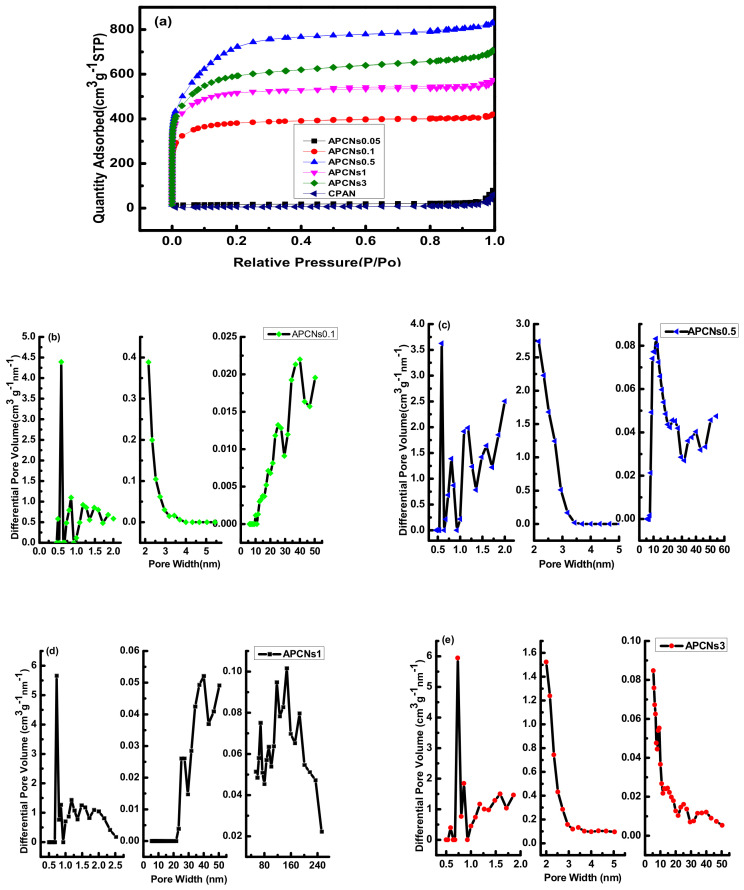
The isotherm adsorption curve and pore size distribution diagram of porous carbon materials with different mass ratios of KOH and OPAN: (**a**) adsorption isotherm curve; mass ratio of: (**b**) 0.1:1; (**c**) 0.5:1; (**d**) 1:1; (e) 3:1.

Figure 11 shows isothermal adsorption curves and pore size distribution of porous carbon materials with different mass ratios of KOH and OPAN. It can be seen from Figure 11a of the N_2_ adsorption and desorption isotherm that the mass ratio is an important factor affecting the porosity of the material. The adsorption isotherm of sample CPAN and APCNs0.05 is concave and has no inflection point. The amount of adsorbed gas increases with the increase in component partial pressure. This is a typical III type adsorption isotherm for non-porous or macropore adsorbents.

Similarly, in the porous carbon materials prepared by the one-step activation method, the structural feature of this graded pore is not only convenient for ion diffusion, but allows the electrolyte to fully contact the surface of the electrolytic material. Therefore, this material with graded pores is suitable for supercapacitor electrode materials [24,25].

Table 5 shows the pore characteristics of porous carbon materials prepared with different KOH and OPAN mass ratios. It can be seen that the specific surface area of CPAN and APCNs0.05 is very small, because CPAN has not been activated to create pores, and the specific surface area of APCNs0.05 does not increase due to the alkali content is too small and the corrosion effect is weak. When the alkali content increases, the specific surface area increases sharply, reaching the maximum when the mass ratio is 0.5. The sample APCNs0.5 has a specific surface area of 2340 m^2^ g^−1^, a total pore volume of 1.29 cm^3^ g^−1^, and has the largest content of mesopores. When the mass ratio of KOH and OPAN is greater than 0.5, the specific surface area decreases as the ratio of mass increases.

The specific surface area increases first and then decreases with the increase in the alkali content. At the beginning, with increase in KOH, micropores are generated as a result of KOH corroding the material. When the alkali content increases further, the pore wall continues to be corroded and may be destroyed, and the micropores expand to form mesopores or macropores, resulting in the reduction in specific surface area.

The adsorption capacity of the porous carbon material prepared by the two-step activation method is higher, with the minimum and maximum adsorption capacities being 713.4 and 1272.3 cm^3^ g^−1^, respectively, while that of the one-step activation method is 494.5 and 919.0 cm^3^ g^−1^. Due to the simultaneous activation and carbonization of the one-step activation method, the reaction is relatively intense, resulting in the continuous corrosion and destruction of the pore structure. At the beginning of the one-step activation method, with the increase in alkali content, the micropores were produced, and specific surface area increase, but with the increase in alkali content, mesopores and macropores appeared, and specific surface area decreased. The two-step activation method obtained the semi-coke morphology by carbonization first, and then the activation reaction was not very intense, the pore structure suffered little corrosion. Therefore, the two-step activation method has more adsorption capacity.

#### 2.2.4. Electrochemical Performance Analysis

**Figure 12:**
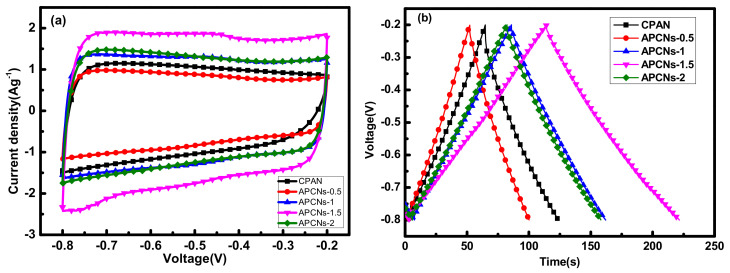
Electrochemical properties of each sample in KOH electrolyte: (**a**) a curve with a scan rate of 10 mV s^−1^ CV; (**b**) a constant current charge and discharge with a current density of 1 A g^−1^.

In Figure 12a, the CV curves of all samples show a rectangular-like shape, which is an ideal double-layer capacitor shape. According to the charge and discharge curve of Figure 12b, the mass-specific capacitances of CPAN, APCNs-0.5, APCNs-1, APCNs-1.5 and APCNs-2 are 98.3, 99.2, 126.8, 178.8 and 127.1 F g^−1^, respectively. The results show that the electrochemical performance of the samples after activation has been improved, and the highest electrochemical performance is in 1.5 h of the activation holding time. Large specific surface area benefits more electrolyte ions to gather at the electrode and electrolyte interface, which is favorable for good electrochemical performance. At the same time, it is reported that nitrogen atoms can increase the wetting between the electrode material and the electrolyte. The pseudo-capacitance is introduced by the Faraday reaction [23,26].

**Figure 13:**
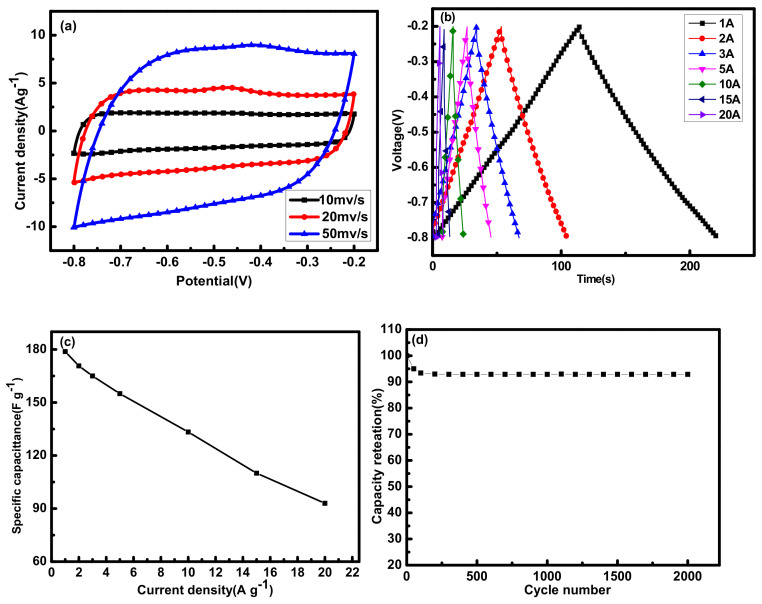
Electrochemical characterization of sample APCNs - 1.5: (**a**) CV curves of different scan rates; (**b**) Constant current charge and discharge curves with current density from 1 A g^−1^ to 20 A g^−1^; (**c**) Constant current charge and discharge line graph with current density from 1 A g^−1^ to 20 A g^−1^; (**d**) Specific capacitance retention rate from current density from 0.5 A g^−1^ to 10 A g^−1^.

The sample APCNs-1.5 was further analyzed for electrochemical performance. In Figure 13a, at a higher scan rate, the CV curve can still maintain a good rectangular-like shape, indicating that the sample APCNs-1.5 has effective charge transfer characteristics [27]. In Figure 13b, the curve presents an isosceles triangle. Figure 13c is the retention rate of the specific capacitance under different current densities. Additionally, the specific capacitance decreases with the increase in current density, which is related to the resistance of ions shutting through nanoscale holes [28]. Figure 13d is the cycle stability diagram of the APCNs-1.5 sample. After 2000 cycles at a current density of 1 A g^−1^, the specific capacitance of the prepared electrode material keeps 92.9%, which indicates that the sample has good cycling stability and is a good electrode material for the supercapacitor.

By comparison the electrical properties of the products for one-step activation method and two-step activation method, it can be seen that the capacitance all reveals good characteristics with the increase in the specific surface. While the capacitance of the sample APCNs-1.5 with 2430 cm^3^ g^−1^ is better than that of the sample 2APCNs-2 with 2830 cm^3^ g^−1^, the reason may be that the electrochemical properties are also related to the pore structure, electrical conductivity and dispersion or chemical composition of other factors.

## 3. Materials and Methods

### 3.1. Preparation of PAN Nanospheres

PAN nanospheres were prepared by soap-free emulsion polymerization. Then, 30 mL AN and 300 mL deionized water were placed in a four-mouth flask at the same time. When the temperature was raised to 75 °C under the protection of nitrogen with continuously stirring, 30 mg KPS (dissolved in deionized water) was added to cause the reaction. The reaction lasted for 6 h and then finished. The polymerized PAN emulsion was freeze-dried and then oxidized under atmosphere (the oxidized PAN was named as OPAN).

### 3.2. Preparation of PAN-Based Porous Carbon Materials

The preparation of PAN-based porous carbon materials by the two-step activation method is divided into two steps. First, OPAN was carbonized at 800 °C to prepare carbon materials of CPAN. Second, CPAN and KOH with a mass ratio of 1:3 was ground evenly and then appropriate amount of deionized water was added and stirred fully. The mixture was dried at 120 °C and then put into a tube furnace for carbonization together with activation at 800 °C. After activation the products were then washed with HCl and deionized water, filtered, and dried to obtain PAN-based porous carbon materials, which is named as 2APCNs-1, 2APCNs-1.5, 2APCNs-2 and 2APCNs-3 (the activation holding time was 1, 1.5, 2 and 3 h, respectively).

In the process of one-step activation method, OPAN was activated directly by KOH with a certain mass ratio to obtain PAN-based porous carbon materials, which is named as APCNs. The effects of activation holding time and weight ratio on the material were studied and named as APCNs-0.5, APCNs-1, APCNs-1.5 and APCNs-2 (activation holding time was 0.5, 1, 1.5 and 2 h, respectively), APCNs0.05, APCNs0.1, APCNs0.5, APCNs1, APCNs3 (0.05:1, 0.1:1, 0.5:1, 1:1, 3:1 by weight of KOH and OPAN, respectively).

### 3.3. Electrode Preparation and Electrochemical Testing

The active substance, conductive agent (carbon black) and binder (PVDF) were mixed at the mass ratio of 8:1:1. N-methyl-2-pyrrolidone was used as the dispersant. The electrode was prepared by using nickel foam as matrix to press the sheet and the electrical performance was tested. The details are the same as those of Yao Long et al. [20].

### 3.4. Material Characterizations

The structure and surface chemical properties of the prepared materials were studied by Quanta 200 scanning electron microscope (SEM, The Dutch FEI), TECNAI F30 transmission electron microscope (TEM, The Dutch FEI), X-ray diffraction (XRD, D8 Advance: Bruker, Germany) and X-ray photoelectron spectroscopy (XPS, Bruker, Germany). The porous properties of the materials were analyzed by 3H-2000PS4 adsorption apparatus from Bruker, Germany.

## 4. Conclusions

PAN polymers could be used to prepare activated porous carbon of high specific surface area and abundant mesopores by both one-step and two-step of activation methods. The specific surface area reached the maximum value with 2430 m^2^ g^−1^ for the one-step activation method and 2830 m^2^ g^−1^ for the two-step activation method, respectively. While the morphology of PAN nanospheres tended to be destroyed in the process of one-step activation and could only be retained when the amount of activating agent KOH was small, which is fairly well as sphere in two-step activation method. The mass-specific capacitances are 178.8 F g^−1^ and 160.2 F g^−1^, respectively, under the current density of 1 A g^−1^. Additionally, after 2000 cycles, the specific capacitance retentions were still good for 92.9% and 91.3%, respectively.

## Figures and Tables

**Table 1:** Elemental analysis of 2APCNs products with different activation holding time.

Element	2APCNs-1	2APCNs-1.5	2APCNs-2	2APCNs-3
C (Atom%)	82.7	88.60	89.18	88.66
N (Atom%)	5.56	5.21	4.05	5.12
O (Atom%)	11.74	6.19	6.77	6.22
O/C (%)	14.2	7.6	7.0	7.1

**Table 2:** Pore characteristics of the materials prepared with different activation holding time in the two-step activation method. (S_BET_ is specific surface area, V_total_ is total pore volume, V_mes_ is mesopore volume and D_p_ is diameter distribution).

Sample	S_BET_ (m^2^ g^−^^1^)	V_total_ (cm^3^ g^−1^)	V_mes_ (cm^3^ g^−^^1^)	D_p_ (nm)
2APCNs-1	1880	1.10	0.71	3.19
2APCNs-1.5	2350	1.50	1.51	3.12
2APCNs-2	2830	2.19	1.97	3.16
2APCNs-3	2670	2.11	1.88	3.19

**Table 3:** Elemental analysis of APCNs products with different activation holding times.

Element	CPAN	APCNs-0.5	APCNs-1	APCNs-1.5	APCNs-2
C (Atom%)	71.29	73.45	80.88	84.16	81.63
N (Atom%)	19.24	4.23	6.24	6.61	4.56
O (Atom%)	0.53	13.29	7.36	6.39	7.31
O/C (%)	7.43	18.09	9.09	7.59	8.95

**Table 4:** Pore properties of porous carbon materials with different activation holding time in one-step activation method.

Sample	S_BET_ (m^2^ g^−1^)	V_total_ (cm^3^ g^−1^)	V_mes_ (cm^3^ g^−1^)	D_p_ (nm)
CPAN	20	0.09	--	--
APCNs-0.5	1080	0.76	0.51	4.85
APCNs-1	1930	1.14	1.13	2.71
APCNs-1.5	2430	1.42	1.14	3.04
APCNs-2	1850	1.09	0.82	3.06

**Table 5:** Pore characteristics of porous carbon materials prepared at different KOH and OPAN mass ratios.

Sample	S_BET_ (m^2^ g^−1^)	V_total_ (cm^3^ g^−1^)	V_mes_(cm^3^ g^−1^)
CPAN	20	0.09	--
APCNs0.05	19	0.10	0.08
APCNs0.1	1170	0.65	0.22
APCNs0.5	2340	1.29	1.04
APCNs1	1590	0.89	0.35
APCNs3	1860	1.09	0.53

## Data Availability

The data presented in this article are available on request from the corresponding author.

## References

[1] Zhai S.L., Karahan H.E., Wang C.J., Pei Z.X., Wei L., Chen Y. (2020). 1D Supercapacitors for Emerging Electronics: Current Status and Future Directions. Adv. Mater..

[2] Thangavel R., Kannan A.G., Ponraj R., Thangavel V., Kim D.W., Lee Y.S. (2018). High-energy green supercapacitor driven by ionic liquid electrolytes as an ultra-high stable next-generation energy storage device. J. Power Sources.

[3] Jost K., Dion G., Gogotsi Y. (2014). Textile energy storage in perspective. J. Mater. Chem. A.

[4] Wang Y.F., Zhang L., Hou H.Q., Xu W.H., Duan G.G., He S.J., Liu K.M., Jiang S.H. (2021). Recent progress in carbon-based materials for supercapacitor electrodes: A review. J. Mater. Sci..

[5] Pan Y.S., Xu K., Wu C.L. (2019). Recent progress in supercapacitors based on the advanced carbon electrodes. Nanotechnol. Rev..

[6] Korivi N.S., Vangari M., Jiang L. (2017). Carbon nanotube nanocomposite-modified paper electrodes for supercapacitor applications. Appl. Nanosci..

[7] Sleptsov V., Diteleva A. (2020). Thin-film technology for creating flexible supercapacitor electrodes based on a carbon matrix. High. Temp. Mater. Process..

[8] Yadav M.S. (2020). Metal oxides nanostructure-based electrode materials for supercapacitor application. J. Nanopart. Res..

[9] Wu D., Li G.N., Fu L.J., Liu Z.L., Wang B.D., Zhang X.H., Qiu D.T., Zhang J.H., Sudduth B., Sun J.M. (2019). Thermodynamics-structure-performance relations of nickel-aluminum layered double hydroxide as supercapacitor electrode materials. Abstr. Pap. Am. Chem. Soc..

[10] Ye J., Shi D.J., Yang Z.K., Chen M.Q. (2018). Interpenetrating Network Hydrogels based on Nanostructured Conductive Polymers for Flexible Supercapacitor. Polym. Sci. Ser. A.

[11] Gang B.J., Zhang F., Li X.L., Zhai B., Wang X.Y., Song Y. (2021). A ulva lactuca-derived porous carbon for high-performance electrode materials in supercapacitor: Synergistic effect of porous structure and graphitization degree. J. Energy Storag..

[12] Zhang G.X., Chen Y.M., Chen Y.G., Guo H.B. (2018). Activated biomass carbon made from bamboo as electrode material for supercapacitors. Mater. Res. Bull..

[13] Kamran U., Choi J.R., Park S.J. (2020). A Role of Activators for Efficient CO_2_ Affinity on Polyacrylonitrile-Based Porous Carbon Materials. Front. Chem..

[14] Zhang X.L., Ma L., Gan M.Y., Fu G., Jin M., Lei Y., Yang P.S., Yan M.F. (2017). Fabrication of 3D lawn-shaped N-doped porous carbon matrix/polyaniline nanocomposite as the electrode material for supercapacitors. J. Power Sources.

[15] Wang C.H., Hsu H.C., Hu J.H. (2014). High-energy asymmetric supercapacitor based on petal-shaped MnO_2_ nanosheet and carbon nanotube-embedded polyacrylonitrile-based carbon nanofiber working at 2 V in aqueous neutral electrolyte. J. Power Sources.

[16] Zhang E.G., Wu M.J., Tang Q.W., Gong Q.J., Sun S.H., Qiao J.L., Zhang L. (2017). Using aminopyrine as a nitrogen-enriched small molecule precursor to synthesize high-performing nitrogen doped mesoporous carbon for catalyzing oxygen reduction reaction. RSC Adv..

[17] Heo Y.J., Zhang Y.F., Rhee K.Y., Park S.J. (2019). Synthesis of PAN/PVDF nanofiber composites-based carbon adsorbents for CO_2_ capture. Composit. Part. B Eng..

[18] Shu Y., Maruyama J., Iwasaki S., Maruyama S., Shen Y., Uyama H. (2017). Fabrication of N-doped and shape-controlled porous monolithic carbons from polyacrylonitrile for supercapacitors. RSC Adv..

[19] Lee B.M., Choi B.S., Lee J.Y., Hong S.K., Lee J.S., Choi J.H. (2021). Fabrication of porous carbon beads from polyacrylonitrile as electrode materials for electric double-layer capacitors. Carbon Lett..

[20] Yao L., Yang G.Z., Han P. (2016). Facile self-templating preparation of polyacrylonitrile-derived hierarchical porous carbon nanospheres for high-performance supercapacitors. RSC Adv..

[21] Liu Y.J., Cao J.Y., Jiang X.H., Yang Y.G., Yu L.M., Yan X.F. (2018). Large scale production of polyacrylonitrile-based porous carbon nanospheres for asymmetric supercapacitors. J. Mater. Chem. A.

[22] Chen G.P., Zhai W.L., Wang Z.H., Yu J.G., Wang F.Q., Zhao Y.N., Li G.D. (2015). Fabrication and supercapacitive properties of hierarchical porous carbon from polyacrylonitrile. Mater. Res. Bull..

[23] Kubota M., Hata A., Matsuda H. (2009). Preparation of activated carbon from phenolic resin by KOH chemical activation under microwave heating. Carbon.

[24] Hesas R.H., Daud W., Sahu J.N., Arami-Niya A. (2013). The effects of a microwave heating method on the production of activated carbon from agricultural waste: A review. J. Anal. Appl. Pyrolysis..

[25] Yahya M.A., Al-Qodah Z., Ngah CW Z. (2015). Agricultural bio-waste materials as potential sustainable precursors used for activated carbon production: A review. Renew. Sust. Energ. Rev..

[26] Li L.X., Song H.H., Chen X.H. (2006). Pore characteristics and electrochemical performance of ordered mesoporous carbons for electric double-layer capacitors. Electrochim. Acta..

[27] Yu X., Kang Y., Park H.S. (2016). Sulfur and phosphorus co-doping of hierarchically porous graphene aerogels for enhancing supercapacitor performance. Carbon.

[28] Ma W.J., Chen S.H., Yang S.Y., Chen W.P., Weng W., Zhu M.F. (2016). Bottom-Up Fabrication of Activated Carbon Fiber for All-Solid-State Supercapacitor with Excellent Electrochemical Performance. ACS Appl. Mater. Int..

